# Gelsolin as a predictor of arteriovenous fistula maturation

**DOI:** 10.1007/s10157-025-02655-2

**Published:** 2025-03-19

**Authors:** Rifat Ozmen, Cihan Uysal, Nevzat Herdem, Funda Ipekten, Inayet Gunturk, Aydin Tuncay, Okan Ozocak, Cevat Yazici, Ismail Kocyigit

**Affiliations:** 1 Department of Cardiovasculary Surgery, Erciyes University School of Medicine, Kayseri, Turkey; 2 Department of Nephrology, Erciyes University School of Medicine, Dede Efendi Street, Köşk, Melikgazi, PC 38030 Kayseri, Turkey; 3 Department of Radiology, Erciyes University School of Medicine, Kayseri, Turkey; 4 Department of Biostatistics, Adiyaman University, Adiyaman, Turkey; 5 Department of Medical Biochemistry, Omer Halisdemir University, Nigde, Turkey; 6 Department of Medical Biochemistry, Erciyes University School of Medicine, Kayseri, Turkey

**Keywords:** Arteriovenous fistula, Maturation, Gelsolin, Hemodialysis, F-actin

## Abstract

**Background:**

Gelsolin is a key regulator of actin filament metabolism and plays a role in tissue remodeling. We evaluated plasma gelsolin (pGSN) in predicting arteriovenous fistula (AVF) maturation.

**Methods:**

Only patients with newly created radiocephalic AVF were included. pGSN and plasma F-actin levels were measured preoperatively. Maturation was defined as an access (cephalic) vein diameter > 5 mm and a fistula blood flow rate > 500 mL/min in ultrasound, 8 weeks after operation.

**Results:**

A total of 68 patients were analyzed with a mean age of 62.6 ± 11.1 years. AVF maturation was identified in 39 patients (57.3%). Mean pGSN level was 4726 (3836–6483) ng/mL in patients with mature AVF and 3237 (2895–4382) ng/mL in patients with immature AVF. pGSN levels were significantly higher (*p* < 0.001) in the mature AVF group. F-actin levels were not significantly different between two groups. pGSN levels positively correlated with fistula blood flows (*r* = 0.326, *p* = 0.007). Multivariate logistic regression analysis revealed that pGSN (p = 0.003) was determined to be an independent risk factor in predicting AVF maturation. Preoperative pGSN levels were significantly predictive of AVF maturation in the ROC analysis. Sensitivity and specificity of pGSN were 82.1% and 58.6%, respectively, with a cut-off value of > 3716 ng/mL and an area under the ROC curve of 0.75 (95% CI: 0.64–0.87, *p* < 0.001).

**Conclusion:**

Current results demonstrated that patients with mature AVFs had significantly higher preoperative pGSN levels compared to those with immature AVFs. Outcomes suggest that pGSN could serve as a predictive biomarker for AVF maturation.

## Introduction

The first preferred vascular access option is the arteriovenous fistula (AVF) in chronic hemodialysis (HD) treatment. A mature AVF should have several features such as sufficient blood flow, a straight and adequate segment for cannulation, and the ability to cannulate repeatedly [1]. The AVF maturation requires appropriate vascular anatomy and an adequate time interval to allow for use [2].

Primary dysfunction and maturation failure are disappointing outcomes of AVF creation. Primary failure is defined as an AVF never ensuring adequate features for dialysis procedures or failing function within three months of use. Early AVF evaluation radiologically at six to eight weeks following creation can provide insight into maturation or additional intervention requirements [3].

Gelsolin belongs to the superfamily of actin-binding proteins and it is a key regulator of actin filament assembly and disassembly. At normal conditions, gelsolin is a highly abundant protein in the circulation. Plasma gelsolin (pGSN) is produced and secreted by virtually every cell type [4]. The cellular actin leakage into the blood occurs after tissue injury, and the purifying actin filaments from plasma by gelsolin help the body recover from the disease [5].

Gelsolin is present in the cell cytoplasm and remains in an inactive state until needed for actin severing. Its function is calcium-dependent and highly sensitive to plasma calcium levels [6]. The binding of calcium ions activates gelsolin by unlocking the three latches that stabilize its inactive form. This calcium binding induces significant conformational changes in the gelsolin structure, which exposes the actin-binding sites and enable its actin-severing activity [7].

More recently, additional roles and cellular functions of gelsolin have been uncovered. Gelsolin plays a critical role in cytoskeletal organization, intracellular signaling, cell morphology, cell migration, and the regulation of cellular junctions. Moreover, gelsolin contributes to wound repair, epithelial-to-mesenchymal transition, and tumor cell invasion [8].

Decreased pGSN levels have been observed in various acute clinical conditions, such as sepsis, myocardial infarction, major trauma, burns, allogeneic stem cell transplantation, and acute liver injury. pGSN levels were inversely related to clinical outcomes in these conditions [9]. Furthermore, increased gelsolin activation has been associated with tumor metastasis in various cancers [10]. Additionally, gelsolin amyloidosis, a rare condition in elderly individuals, has been linked to nephrotic syndrome and progressive kidney disease [11].

The current investigation focuses on the potential biomarker role of pGSN in AVF maturation. Our aim is to evaluate preoperative pGSN levels as predictors of AVF maturation in the end-stage kidney disease (ESKD) population.

## Materials and methods

### Study design

This a cross-sectional study was conducted at a single center. Only adult patients (age ≥ 18 years) were included. The key inclusion criterion was an estimated glomerular filtration rate (eGFR) below 15 ml/min/1.73 m^2^ and a newly created AVF prior to the initiation of chronic HD treatment. Patients undergoing chronic HD before this study were excluded. Patients with malignancies, heart failure, and acute illnesses like infections during the perioperative period were also excluded.

### AVF creation

The same cardiovascular surgical team conducted all AVF creation procedures. All AVFs were created as radiocephalic fistulas at the forearm location. Before the AVF creation, all patients underwent a physical examination to assess their suitability for AVF. For patients requiring immediate HD initiation, a dialysis catheter (temporary or tunneled) was inserted in the contralateral position to the AVF. After preparing the radial artery and cephalic vein for anastomosis under local anesthesia, end-to-side anastomosis was performed to create the radiocephalic fistula. Patients with early primary failure of the AVF after the operation were not included in the follow-up period. Additionally, patients who required further interventional assistance for AVF maturation during the follow-up were excluded from the study. Finally, no anticoagulant or antiaggregant drugs were used to improve the primary patency of the AVF in the postoperative period.

### AVF maturation assessment

**Fig. 1:**
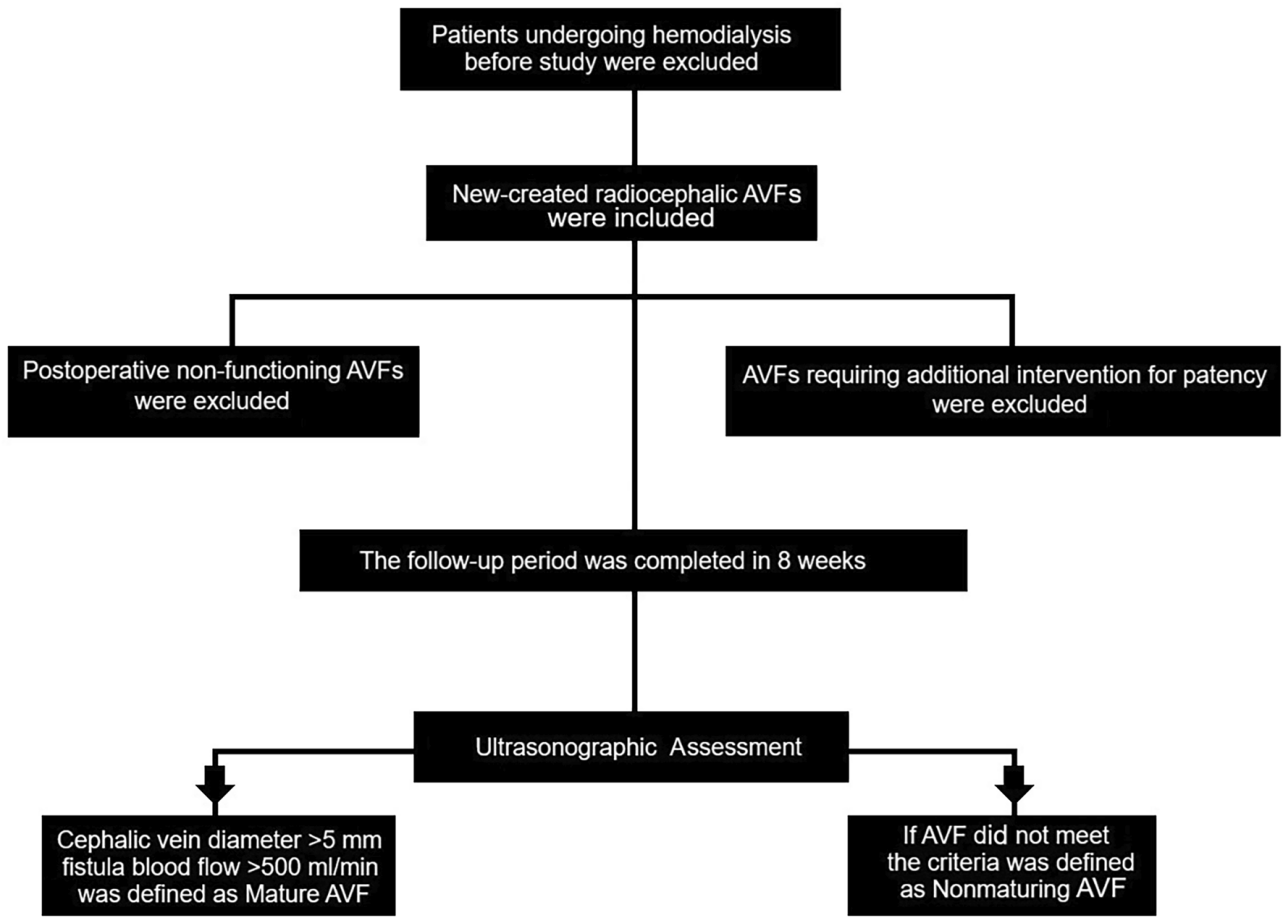
Algorithm for vascular evaluation and patient selection

AVF maturation was assessed through duplex ultrasonography (USG) and physical examination. USG monitoring was conducted 8 weeks post-operation. The diameters of the access vessel (cephalic vein) and feeding artery (radial artery), as well as the fistula blood flow rate (measured 5 cm proximal to the anastomosis), were evaluated using duplex USG (Logiq S7/Expert, GE Healthcare; Milwaukee, WI, USA). All assessments were performed by the same radiologist. A mature AVF was defined as having a cephalic (access) vein diameter > 5 mm and a fistula blood flow > 500 ml/min. The algorithm for vascular evaluation and patient selection is shown in Fig. 1.

### Gelsolin and F-actin measurement

Blood samples for the measurement of plasma gelsolin and F-actin levels were collected before the AVF operation. Venous blood samples were centrifuged for 10 min at 5000 rpm (NF 400 centrifuges, Turkey). The samples were stored at − 80 °C until analysis.

Gelsolin levels were determined using a USCNK brand ELISA kit (Cloude-Clone Corp, 23,603 W. Fernhurst Dr., Unit 2201, Katy, TX 77494, USA, Catalog No: SEA372Hu) according to the manufacturer’s instructions and were expressed as ng/mL. F-actin levels were measured using a Cusabio brand ELISA kit (CUSABIO Technology LLC, 7505 Fannin St Ste 610-322 Houston, TX 77054, USA, Catalog No: CSB-E13678h) according to the manufacturer’s instructions and were also expressed as ng/mL.

### Statistical analysis

Histograms and Q-Q plots were examined, and the Shapiro–Wilk test was applied to assess data normality. The Levene test was used to evaluate variance homogeneity. To compare differences between groups, either independent-samples *t*-tests or Mann–Whitney U tests were employed for continuous variables. The relationship between quantitative data was analyzed using Spearman correlation analysis. ROC analysis was performed to identify the predictive ability of gelsolin and F-actin markers, and the area under the ROC curves was calculated with 95% confidence intervals. Univariate and multiple binary logistic regression analysis were applied to identify the risk factors of AVF maturation. For each marker, cut-off values were determined using the Youden index. Using these cut-off values, sensitivity, specificity, positive predictive values, and negative predictive values were calculated with 95% confidence intervals for each marker. Analyses were conducted using R 4.3.2 (www.r-project.org). A p-value less than 0.05 was considered statistically significant.

## Results

In this study, a total of 68 patients were analyzed, with a mean age of 62.6 ± 11.1 years. Fifty-two patients (76.4%) underwent only AVF creation as their first HD access. A dialysis catheter was inserted concurrently with AVF creation in 16 patients (23.6%) due to immediate HD initiation. Diabetes mellitus (DM) was prevalent in 35.3% of the patients (*n* = 24) and was identified as the most common cause of kidney disease.

Mature AVF was identified in 39 patients (57.3%). The median fistula blood flow rate was 690.0 (655.0–780.0) mL/min in patients with mature AVF, compared to 200.0 (145.0–375.0) mL/min in those with immature AVF. The mean cephalic vein diameter was 5.3 (5.2–5.7) mm in patients with mature AVF and 2.2 (1.8–4.2) mm in patients with immature AVF. The median radial artery diameter was 4.1 (3.6–4.7) mm in patients with mature AVF and 2.6 (2.2–3.3) mm in those with immature AVF.

The average age was 59.5 ± 10.9 years in the mature AVF group and 66.7 ± 10.1 years in patients with immature AVF (*p* = 0.007). AVF maturation frequencies were not significantly different between th two genders (*p* = 0.624). The baseline characteristics and clinical features of patients are summarized in Table 1. In the laboratory analysis, no parameters showed statistically significant differences between the groups. A summary of the laboratory parameters is provided in Table 2.

**Table 1:** Baseline characteristics and clinical features of patients

Features	Mature AVF (*n* = 39)	Immature AVF (*n* = 29)	*p* value
Age (years)	59.5 ± 10.9	66.7 ± 10.1	**0.007**
BMI (kg/m^2^)	24.7 (22.3–28.5)	24.1 (22.4–27.6)	0.744
Gender			0.624
Male	29 (74.3%)	20 (68.9%)	
Female	10 (25.7%)	9 (31.1%)	
Etiological disease			0.293
Diabetes mellitus	10 (37.6%)	14 (48.3%)	
Hypertension	11 (20.8%)	8 (27.6%)	
Glomerular diseases	6 (14.3%)	3 (10.3%)	
Urological problems	5 (7.8%)	3 (10.3%)	
ADPKD	3 (7.8%)	1 (3.4%)	
Unknown	4 (11.7%)	0 (0%)	
Hypertension	23 (79.3%)	24 (82.7%)	0.192
Obesity	4 (13.8%)	8 (20.5)	0.691
Coronary arter disease	5 (12.8%)	12 (41.4%)	**0.008**
Peripheral vascular disease	1 (2.6%)	3 (10.3%)	0.204
Requirement of immediate HD	10 (25.6%)	6 (20.7%)	0.852
Smoking	3 (3.9%)	4 (5.2%)	0.056

Values are expressed as mean ± standard deviation, median (1st-3rd quartiles) and n(%). *BMI* Body mass index, *HD* Hemodialysis, *ADPKD* Autosomal dominant polycystic kidney disease, *AVF* Arteriovenous fistula

Statistically significant *p*-values are shown in bold

**Table 2:** Comparison of laboratory parameters according to the AVF maturation groups

Parameters	AVF Maturation		*p*
	Present (*n* = 39)	Absent (*n* = 29)	
Glucose (mg/dL)	120.0 (93.0–174.0)	132.0 (106.0–167.0)	0.270
BUN (mg/dL)	45.6 (37.3–61.5)	44.3 (34.4–60.0)	0.620
Creatinine (mg/dL)	4.6 (3.8–6.0)	3.8 (3.3–5.3)	0.074
Uric acid (mg/dL)	6.3 (4.7–7.1)	5.6 (3.6–6.7)	0.231
Protein (g/dL)	6.7 ± 0.9	6.4 ± 0.7	0.246
Albumin (g/dL)	3.7 ± 0.5	3.7 ± 0.5	0.829
Sodium (mmol/L)	137.6 ± 3.7	137.8 ± 2.69	0.879
Potassium (mmol/L)	4.5 ± 0.7	4.5 ± 0.5	0.957
Calcium (mg/dL)	8.7 ± 0.8	8.6 ± 0.7	0.596
Phosphate (mg/dL)	4.1 (3.1–5.0)	4.4 (3.6–5.0)	0.664
CaxP (mg^2^/dL^2^)	36.5(27.0–46.7)	35.8 (29.3–42.7)	0.990
PTH (pg/mL)	204.0 (120.5–276.3)	207.0 (93.5–281.5)	0.606
AST (IU/mL)	14.0 (11.0–18.0)	13.0 (10.0–20.5)	0.576
ALT (IU/mL)	10.0 (8.0–14.0)	10.0 (9.0–12.5)	0.995
Hemoglobin (g/dL)	10.7 ± 1.8	10.5 ± 1.6	0.590

Values are expressed as mean ± standard deviation, median (1st-3rd quartiles). *BUN* Blood urea nitrogen, *CaxP* Calcium phosphate product. *AVF* Arteriovenous fistula, *PTH* Parathyroid hormone

The average pGSN level was 4301 (3204–5376) ng/mL in all patients, while the average F-actin level was 3.4 (2.0–4.6) ng/mL. The pGSN level was significantly higher (*p* < 0.001) in the mature AVF group, with values of 4707 (3897–6237) ng/mL in patients with mature AVF and 3236 (2895–4382) ng/mL in those with immature AVF. The average F-actin level was 3.2 (1.6–4.4) ng/mL in patients with mature AVF and 3.6 (2.4–5.6) ng/mL in patients with immature AVF. There was no statistically significant difference in plasma F-actin levels between the groups (*p* = 0.132).

**Fig. 2:**
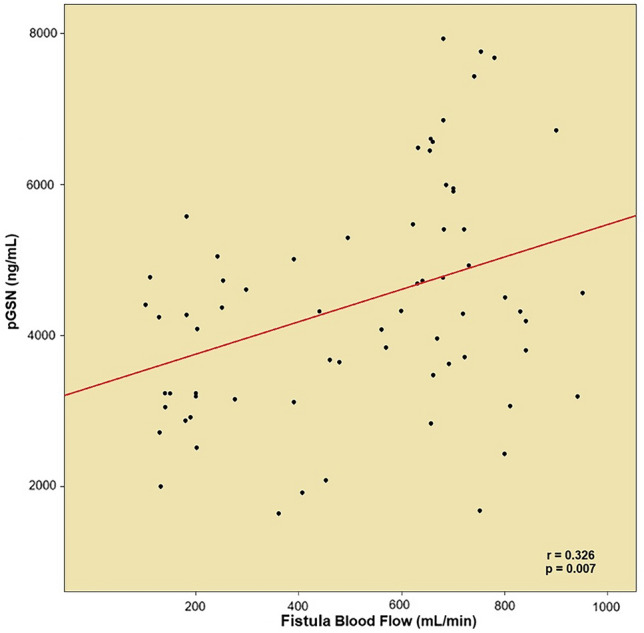
Correlation analysis of pGSN and access vein blood flows in scatter plot

pGSN levels positively correlated with fistula blood flow rates (*r* = 0.326, *p* = 0.007), and radial artery diameters (*r* = 0.281, *p* = 0.020), however, no correlation was identified with cephalic vein diameters (*p* = 0.069). Results are shown in Fig. 2.

**Fig. 3:**
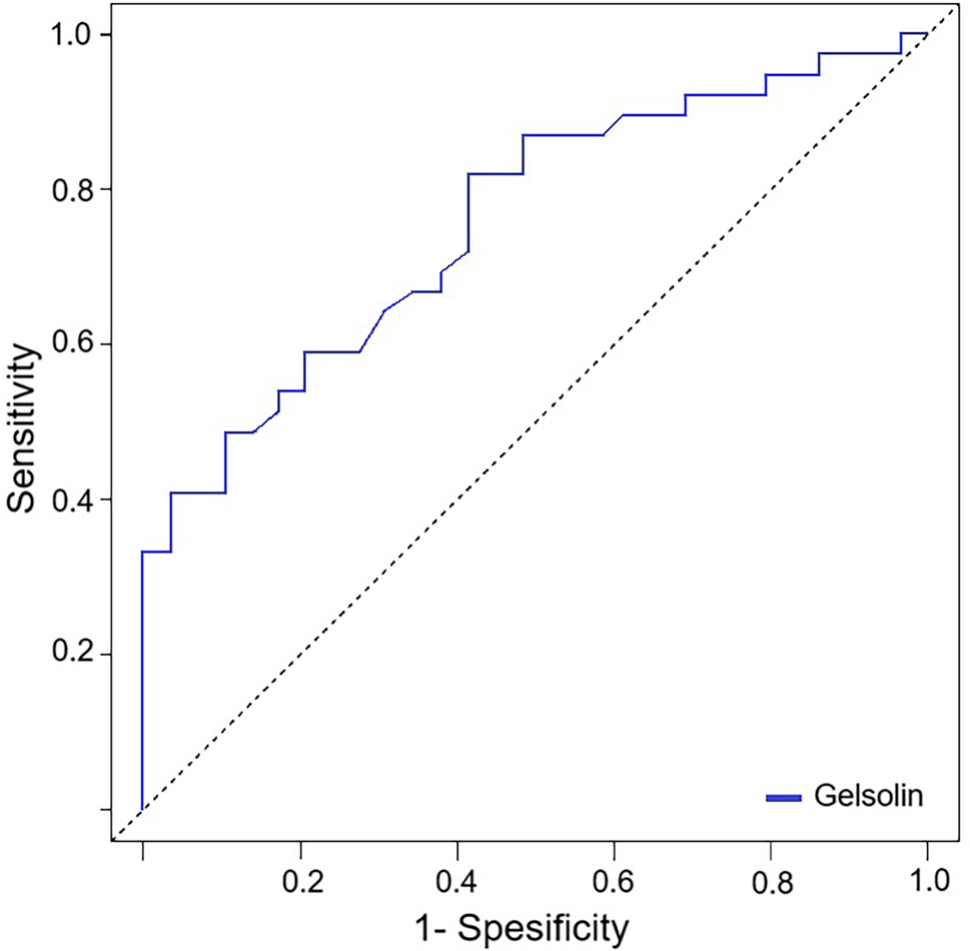
ROC curves of pGSN in predicting AVF maturation

The pGSN level was a significant predictor in ROC analysis for AVF maturation, as shown in Fig. 3. The probability of pGSN for predicting AVF maturation was found to be 75.95%. The sensitivity of pGSN was 82.1%, specificity was 58.6%, positive predictive value was 72.7%, and negative predictive value was 70.8%. The probability of the F-actin biomarker predicting AVF maturation was found to be 63.01%, but this was not statistically significant (*p* = 0.056). The results are summarized in Table 3.

**Table 3:** The results of the ROC curve analysis

Parameters	ROC statistics		Diagnostic statistics			
	AUC (%95 CI)	*p value*	SEN (%95 CI)	SPE (%95 CI)	PPV (%95 CI)	NPV (%95 CI)
pGSN (> 3716 ng/mL)	75.9 (64.66–87.24)	** < 0.001**	82.1 (66.5–92.5)	58. (38.9–76.5)	72.7 (54.6–87.7)	70.8 (51.3–84.8)
F-actin (> 4.6 ng/mL)	63.0 (49.67–76.51)	0.056	35.9 (21.2–52.8)	89.7 (72.6–97.8)	82.4 (58.9–90.3)	21.0 (33.3–84.3)

*AUC* Area under the curve, *ROC* Receiver Operating Characteristics, *SEN* Sensitivity, *SPE* Specificity, *PPV* Positive predictive value, *NPV* Negative predictive value, *pGSN* plasma gelsolin level

Statistically significant *p*-values are shown in bold

For the AVF maturation, univariate and multiple binary logistic regression analysis results are shown in Table 4. The results revealed that age (*p* = 0.019), coronary artery disease (*p* = 0.040), and pGSN (*p* = 0.003) were determined to be independent risk factors in predicting AVF maturation. The odds ratio of pGSN was 6.48 (2.15–19.50).

**Table 4:** Logistic regression analysis in identifying the risk factors on the AVF maturation

Variables	Univariate		Multiple	
	OR (95% CI)	*p* value	OR (95% CI)	*p* value
Age	0.93 (0.88–0.98)	**0.007**	0.92(0.86–0.99)	**0.019**
Gender (male)	1.31 (0.45–3.79)	0.624	–	–
Protein	1.02 (0.66–1.56)	0.945	–	–
Albumin	0.97 (0.40–2.36)	0.947	–	–
Ca*P	0.99 (0.95–1.04)	0.692	–	–
Diabetes	2.71 (0.97–7.53)	0.056	–	–
Hypertension	2.40 (0.79–7.24)	0.122	–	–
Obesity	1.61 (0.44–5.98)	0.475	–	–
Smoking	3.11 (1.10–8.82)	**0.033**	–	–
Coronary artery disease	4.80 (1.45–15.85)	0.010	4.52(1.07–19.15)	**0.040**
Immideate HD	1.32 (0.42–4.18)	0.635	–	–
Gelsolin	6.48 (2.15–19.50)	** < 0.001**	7.68(2.01–29.40)	**0.003**
F-actin	4.33 (1.10–17.02)	**0.036**	–	–

*OR* Odds ratio, *CI* Confidence interval, Ca*P calcium phosphate products, *HD* Hemodialysis. *AVF*: Arteriovenous fistula. Significant *p*-values are shown in bold

## Discussion

These results suggest that pGSN levels could serve as a biomarker for AVF maturation in ESKD. Gelsolin has previously been identified as a prognostic factor in various cardiovascular diseases. In this study, we focused on AVF maturation, a vascular remodeling process. We did not find any reports on this topic in the literature. Therefore, the current research is both significant and enlightening in this regard.

Our results revealed that patients with mature AVF had higher preoperative pGSN levels compared to those with immature AVF, with values of 4726 ng/mL and 3236 ng/mL, respectively. Furthermore, we identified pGSN as a predictive biomarker for AVF maturation.

Gelsolin is an abundant protein in circulation, secreted by nearly all human cells. Its primary function is to sever F-actin molecules in the bloodstream. In addition to this, many other functions of gelsolin have been demonstrated. It plays critical roles in cellular motility, growth, cytoskeletal organization, programmed cell death, and the regulation of intercellular junctions through actin metabolism [8–10]. Thus, gelsolin is a vital factor for normal development and tissue remodeling.

The clinical and therapeutic value of gelsolin has been well-established in animal models and in patients with various diseases. Mikami et al. demonstrated that muscle cell relaxation was impaired in gelsolin knockout mice [11]. In another study, it was reported that intensive care patients with lower pGSN levels, compared to healthy individuals, experienced higher mortality rates and longer hospitalizations. Additionally, pGSN levels increased in patients recovering from illnesses [12].

In the literature, several studies have reported a relationship between pGSN levels and vascular disease outcomes. Gelsolin levels were significantly decreased in cardiovascular diseases and myocardial infarction. Lower pGSN levels have been identified as a risk factor for ascending aortic aneurysm in bicuspid aortic valve disease [13]. Additionally, reduced pGSN levels have been associated with a higher risk of aortic vascular calcification in HD patients [14]. Li, Guo Hua, et al. demonstrated the proliferative effect of gelsolin in mice, suggesting that gelsolin may contribute to cardiac remodeling and the progression of heart failure following experimental myocardial infarction [15]. These findings highlight the role of gelsolin in vascular structural alterations. Shi et al. reported that decreased pGSN levels were associated with acute kidney injury and poor clinical outcomes after cardiopulmonary bypass surgery [16].

Furthermore, the gelsolin molecule has been investigated in experimental studies for therapeutic purposes. The thrombo-protective capability of exogenous gelsolin was demonstrated in models of ferric chloride-induced thrombosis of the carotid artery and thrombin-induced acute pulmonary thromboembolism in mice [17]. Additionally, gelsolin replacement therapy has proven effective in some animal models, such as those involving sepsis [18].

The explanation for the current results is not entirely clear. However, it can be inferred that increased pGSN levels are associated with a successful vascular remodeling process. The actin cytoskeleton is a crucial network of filaments found in all cells, playing an important role in regulating cellular activities. It is essential for effective tissue remodeling and wound repair. Therefore, gelsolin serves as a key regulator of tissue healing at different stages [19]. Additionally, actin filaments are involved in muscle contraction and relaxation, and smooth muscle relaxation and vessel dilation are critical steps in the AVF maturation process. Gelsolin may contribute to the success of this process; however, we believe this contribution is independent of F-actin severing in the bloodstream. We hypothesize that positive outcomes are associated with the successful assembly of actin filaments due to increased pGSN levels, as plasma F-actin levels were not significantly correlated with maturation outcomes.

The evolution of AVF can be delineated through four distinct stages: fistula creation, maturation, initial clinical deployment, and sustained clinical utilization. In the context of USG criteria, maturation is typically defined by a minimum access vessel diameter of ≥ 5 mm and a blood flow rate of ≥ 500 mL/min [1]. The maturation period for AVF generally spans at least one month, although this duration may extend up to six months for patients with smaller vessels. Interventions may be necessary to facilitate the maturation process [20]. Early thrombosis is a significant contributor to the primary failure of newly created AVFs. In a study of 877 patients, early fistula thrombosis was responsible for 15.6% of primary fistula failure cases [21].

The physiology of AVF maturation has been extensively investigated. Following AVF creation, blood is redirected into the vein, leading to a significant increase in blood flow through the arterial feeder due to reduced outflow resistance. This elevated venous blood flow triggers adaptive changes in vessel structure. Successful AVF maturation requires both functional and structural adjustments in the inflow artery and outflow vein. The increased venous flow results in a proportional elevation in vessel wall shear stress, which promotes endothelium-dependent vasodilation in both the artery and vein. This process is primarily mediated by nitric oxide and matrix metalloproteinases, leading to vasodilation and suppression of neointimal hyperplasia [22].

During the second phase of AVF development, a significant challenge arises when maturation fails, defined as the inability of an AVF to become suitable for HD procedures or its failure within three months of creation. According to a multicenter study, the incidence of non-maturing AVFs was reported to be 17%. The primary cause of maturation failure typically involves anatomical issues, such as vascular stenosis, which can be identified through clinical examination and confirmed via imaging modalities [23]. AVF-related complications are classified into inflow and outflow problems.

Multiple factors influence the functional maturation of AVFs. Factors associated with AVF maturation include age, gender, obesity, urea levels, electrolytes, blood pressure, and smoking [24]. Additionally, DM has been identified as a significant negative predictor of venous remodeling in a retrospective study [25].

A meta-analysis of 13 cohort studies, including 11 retrospective analyses, demonstrated a higher primary failure rate and poorer patency of radiocephalic AVFs in elderly individuals [26]. In another study, Kim et al. revealed that a link higher age and intima-media width in microscopic analysis. Also, Increased intima-media thickness leads to a loss of vascular elasticity and luminal narrowing, ultimately contributing to greater arterial rigidity [27]. Consequently, the AVF failure rate is higher in elderly patients. Likewise, the average age was 66.7 years in patients with immature AVF, whereas it was 59.5 years in patients with mature AVF in our cohort.

In this study, we included only patients with radiocephalic AVFs to ensure individual similarity and minimize factors that may affect AVF maturation. The anatomical location of vascular access is an important variable in the maturation of AVFs, as maturation rates vary across different vessel locations. One study involving 808 HD patients reported that the maturation time required to avoid catheter use was significantly shorter for upper arm fistulas [28]. Additionally, we excluded cases that required interventional assistance, such as thrombectomy due to acute thrombosis, from the follow-up. We believed that a pure maturation process would not be achievable in these cases.

In conclusion, this study addressed a significant issue for patients with ESKD requiring dialysis treatment: the maturation of AVFs. Furthermore, these findings represent the first investigation into this topic. The current results may serve as a source of inspiration for future experimental or therapeutic studies on gelsolin in relation to AVF maturation.
